# Supplementary figures and images for: Transcriptional and genomic parallels between the monoxenous parasite Herpetomonas muscarum and Leishmania
Source: PLoS Genet. 2019 Nov 11;15(11):e1008452. doi: 10.1371/journal.pgen.1008452 (PMC6872171; doi:10.1371/journal.pgen.1008452)

A

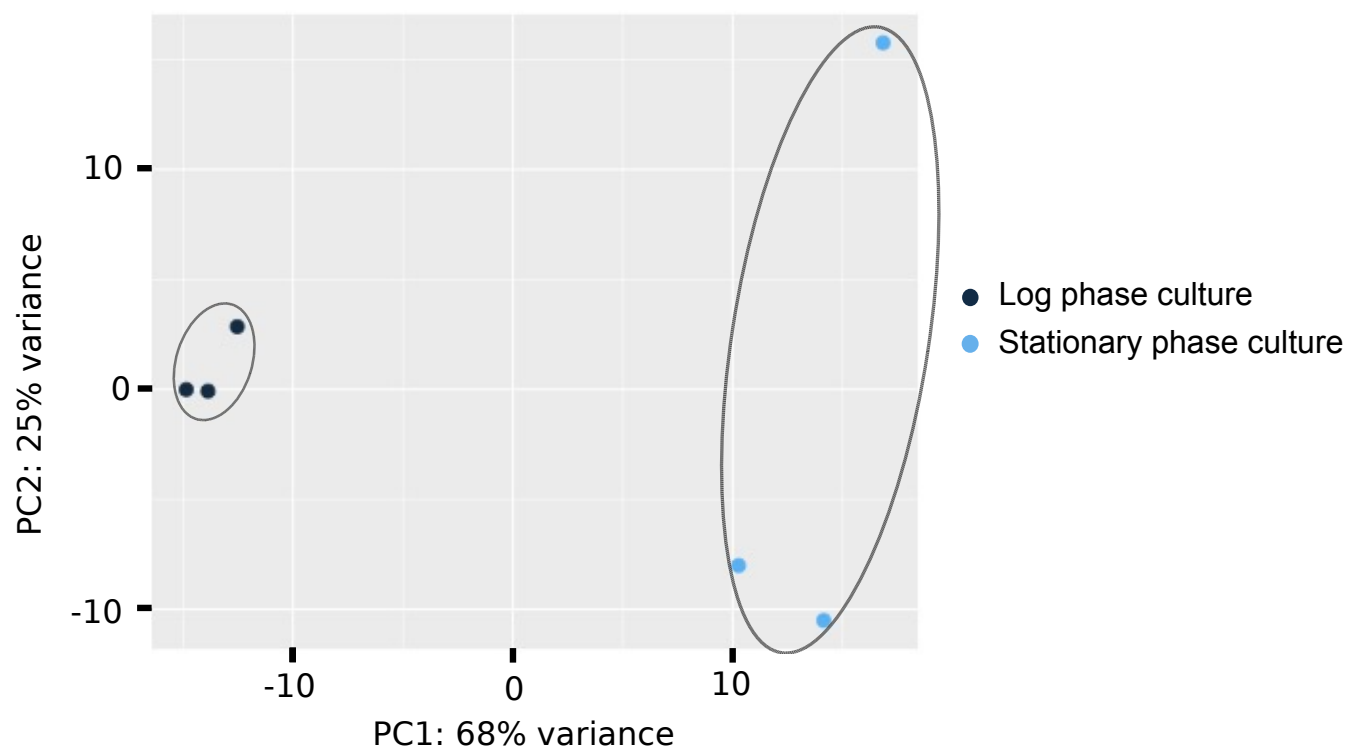

Supplement: S2 Fig — (A). There are two clear sample groupings (circled) which correspond to RNA each condition (n = 3 per condition). Dark blue = log phase samples and light blue = stationary phase samples. (PDF) [file pgen.1008452.s002.pdf]
